# Analyzing the safety of the parasiticide fungus *Mucor circinelloides*: first insights on its virulence profile and interactions with the avian gut microbial community

**DOI:** 10.1128/spectrum.04078-23

**Published:** 2024-03-27

**Authors:** João Lozano, Eva Cunha, Cristina Almeida, Mónica Nunes, Ricardo Dias, Eduardo Vicente, Daniela Sebastião, Sérgio Henriques, Luís Madeira de Carvalho, Adolfo Paz-Silva, Manuela Oliveira

**Affiliations:** 1 CIISA – Centre for Interdisciplinary Research in Animal Health, Faculty of Veterinary Medicine, University of Lisbon, Lisbon, Portugal; 2 Associate Laboratory for Animal and Veterinary Sciences (AL4AnimalS), Lisbon, Portugal; 3 Exoclinic – Clínica Veterinária de Aves e Exóticos, Miraflores, Portugal; 4 Biosystems and Integrative Sciences Institute, Faculty of Sciences, University of Lisbon, Lisbon, Portugal; 5 Castelo de São Jorge, EGEAC – Empresa de Gestão de Equipamentos e Animação Cultural, Lisbon, Portugal; 6 Quinta da Galeana, Ovinos do Futuro, Lda., Nadrupe, Portugal; 7 Control of Parasites Research Group (COPAR, GI-2120), Department of Animal Pathology, Faculty of Veterinary, University of Santiago de Compostela, Lugo, Spain; 8 cE3c – Centre for Ecology, Evolution and Environmental Changes, Faculdade de Ciências, Universidade de Lisboa, Lisbon, Portugal; 9 CHANGE – Global Change and Sustainability Institute, Faculdade de Ciências, Universidade de Lisboa, Lisbon, Portugal; Texas A&M University, College Station, Texas, USA

**Keywords:** avian parasitology, parasiticide fungi, virulence factors, microbiome, mycobiome

## Abstract

Parasiticide fungi are considered an accurate, sustainable, and safe solution for the biocontrol of animal gastrointestinal (GI) parasites. This research provides an initial characterization of the virulence of the native parasiticide fungus *Mucor circinelloides* (FMV-FR1) and an assessment of its impact on birds’ gut microbes. The genome of this fungus was sequenced to identify the genes coding for virulence factors. Also, this fungus was checked for the phenotypic expression of proteinase, lecithinase, DNase, gelatinase, hemolysin, and biofilm production. Finally, an *in vivo* trial was developed based on feeding *M. circinelloides* spores to laying hens and peacocks three times a week. Bird feces were collected for 3 months, with total genomic DNA being extracted and subjected to long-read 16S and 25S-28S sequencing. Genes coding for an iron permease (FTR1), iron receptors (FOB1 and FOB2), ADP-ribosylation factors (ARFs) (ARF2 and ARF6), and a GTPase (CDC42) were identified in this *M. circinelloides* genome. Also, this fungus was positive only for lecithinase activity. The field trial revealed a fecal microbiome dominated by Firmicutes and Proteobacteria in laying hens, and Firmicutes and Bacteroidetes in peacocks, whereas the fecal mycobiome of both bird species was mainly composed of Ascomycetes and Basidiomycetes fungi. Bacterial and fungal alpha-diversities did not differ between sampling time points after *M. circinelloides* administrations (*P* = 0.62 and *P* = 0.15, respectively). Although findings from this research suggest the lack of virulence of this *M. circinelloides* parasiticide isolate, more complementary *in vitro* and *in vivo* research is needed to conclude about the safety of its administration to birds, aiming at controlling their GI parasites.

**IMPORTANCE:**

A previous study revealed that the native *Mucor circinelloides* isolate (FMV-FR1) can develop parasiticide activity toward coccidia oocysts, one of the most pathogenic GI parasites in birds. However, ensuring its safety for birds is of utmost importance, namely by studying its virulence profile and potential effect on commensal gut microbes. This initial study revealed that although this *M. circinelloides* isolate had genes coding for four types of virulence factors—iron permease, iron receptors, ADP-ribosylation factors, and GTPase—and only expressed phenotypically the enzyme lecithinase, the administration of its spores to laying hens and peacocks did not interfere with the abundances and diversities of their gut commensal bacteria and fungi. Although overall results suggest the lack of virulence of this *M. circinelloides* isolate, more complementary research is needed to conclude about the safety of its administration to birds in the scope of parasite biocontrol programs.

## INTRODUCTION

Parasiticide fungi are a functional group of microorganisms known for their ability to destroy the exogenous forms of gastrointestinal (GI) parasites, namely coccidia oocysts, and also helminth eggs and larvae (1). Fungi like *Duddingtonia flagrans*, *Arthrobotrys oligospora* and *Monacrosporium thaumasium*, develop trapping structures to immobilize and destroy nematodes’ infective larvae (larvicidal fungi), whereas *Mucor circinelloides* and *Pochonia chlamydosporia*, destroy coccidia oocysts and helminth eggs (ovicidal fungi) (2–4). The majority of research on this topic has been performed in ruminants and horses (5–8) and more recently extended to the control of parasites affecting other animal hosts, namely dogs, birds, and captive wild animals (4, 9, 10).

Considering that some fungi of the order Mucorales, such as *M. circinelloides*, are commonly linked to opportunistic infections, which may lead to mucormycosis in immunocompromised humans and animals (11), studying their safety is a mandatory step to implement this parasite biological control approach at field level.

Birds’ native gut microbiota (i.e., bacterial community) is mainly composed of bacteria of the phyla Firmicutes, Proteobacteria, and Bacteroidetes, with its diversity and relative abundances being influenced by several endogenous and exogenous factors, such as diet, age, sex, health status, and environmental microorganisms (12). Although most studies are still focused on animal gastrointestinal (GI) bacteria, new information is being recorded for animals’ enteric fungal communities (i.e., mycobiota), revealing that the Ascomycota and Basidiomycota phyla are the most dominant in the GI tract of poultry (13, 14). Since parasiticide fungi spores are often administered orally to animals and then pass through the GI tract and are finally expelled with feces to the environment, where they develop larvicidal or ovicidal activities toward parasitic forms (1, 2), ensuring that fungal formulations do not disturb the native gut micro- and mycobiota is also crucial for maintaining intestinal homeostasis, which is, to our knowledge, a topic not yet studied.

The current research aimed to perform an initial characterization of the virulence profile of the native parasiticide fungus *Mucor circinelloides* (FMV-FR1) and assess its potential influence on birds’ GI native bacterial and fungal communities in the scope of a larger project aiming at controlling peacocks’ coccidia.

## RESULTS

### *M. circinelloides* (FMV-FR1) virulence factors

The complete analysis of *M. circinelloides* (FMV-FR1) genome revealed six predicted genes coding for virulence factors, above the 80% identity cut-off, namely the gene FTR1, which encodes for an iron permease (protein ID: I1BRD6; *Rhizopus arrhizus*, 83.98% identity; PHI database), the genes FOB1 (protein ID: I1BW02; *Rhizopus arrhizus*, 83.07% identity; PHI database) and FOB2 (protein ID: I1CCV9; *Rhizopus arrhizus*, 86.67% identity; PHI database), which encode for iron receptors, the genes ARF2 (protein ID: Q5AND9; *Candida albicans*, 90.06% identity; PHI database) and ARF6 (protein ID: G4N9S6; *Magnaporthe oryzae*, 80.45% identity; PHI database), which encode for ADP-ribosylation factors, as well as the gene CDC42 (protein ID: Q2PBY8_CLAPU; *Claviceps purpurea*, 86.84% identity; DFVF database), which encodes for a Rho-like GTPase.

All other common Mucorales virulence factors, such as iron ferroxidase (FET3 gene), spore coat proteins (COTH2 and COTH3 genes), protein kinase A (PKAR gene), endonucleases (DCL1 gene), 14-α sterol demethylases (CYP51 gene), transcription factors (ATF1 gene), heteromeric G-protein beta subunit (GPB1 gene), siderophores (ARN1, SREA, BIR1, and AFT1 genes) and chitinase (GH18), were detected under the threshold of 80% identity (Table S1).

**Fig 1:**
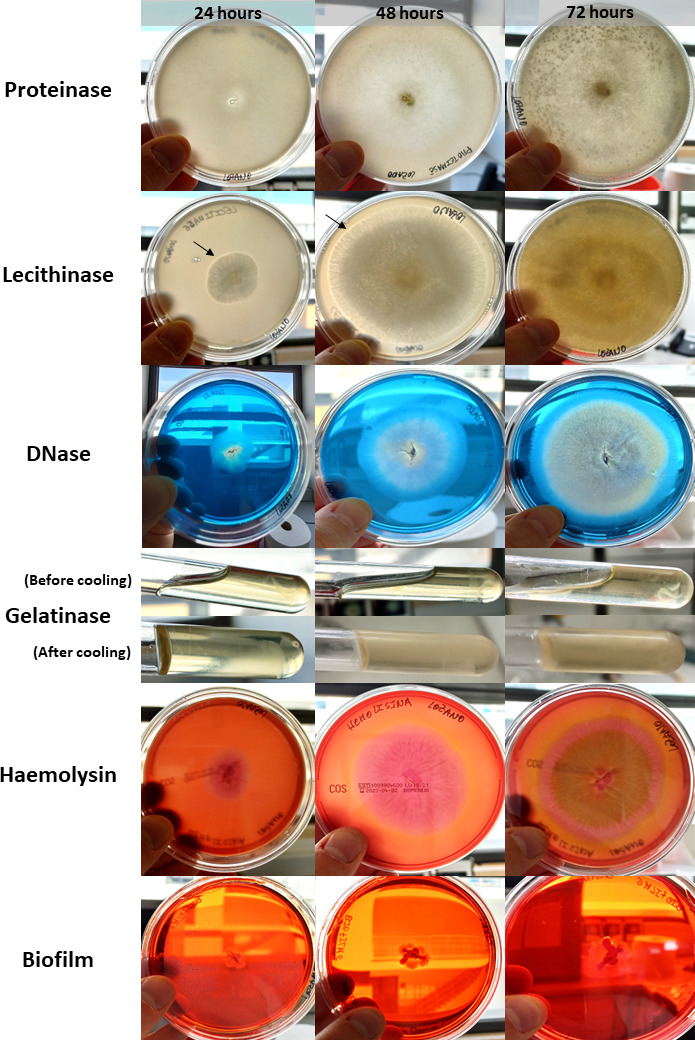
Phenotypic expression of six virulence factors by *M. circinelloides* (FMV-FR1). Positive results for lecithinase activity are revealed by a clearing halo surrounding the fungal colony, observed after 24 and 48 h of incubation (black arrows). This isolate tested negative for all other virulence factors.

Regarding the phenotypic expression of virulence factors, based on the culture media available for this assay, this *M. circinelloides* isolate was found to be positive only for lecithinase activity. Negative results were obtained for the other virulence factors tested, with fungal growth not leading to any media changes (Fig. 1).

**Fig 2:**
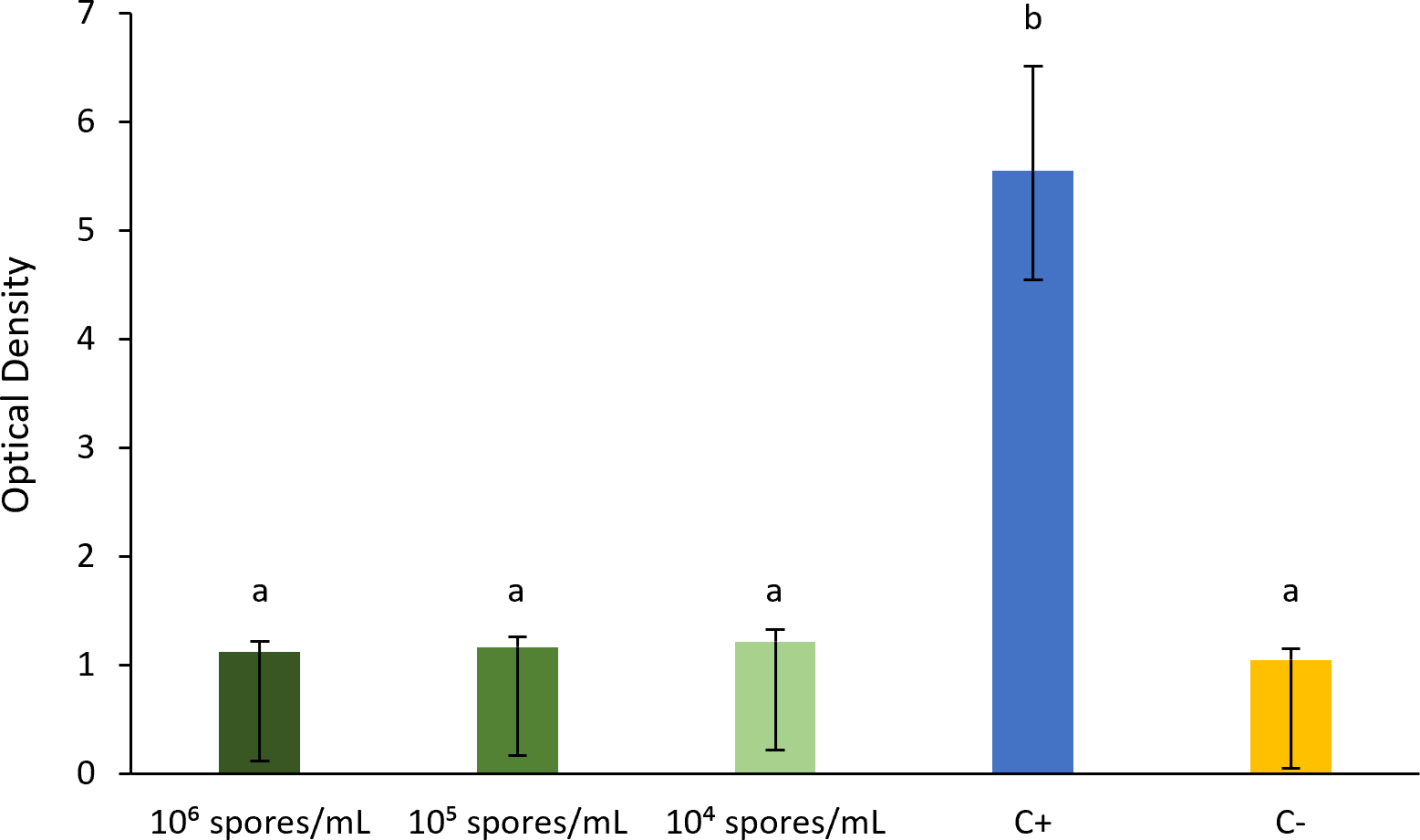
Optical density (OD) results (mean ± standard error) obtained for test wells containing rRBCs and each fungal concentration (green bars), and for positive (with rRBCs +Triton 1%) and negative control wells (with PBS) (C+ and C−, respectively). OD results were measured by absorbance at 450 nm. Bars sharing the same superscript letter correspond to non-significant differences (*P* > 0.05).

Also, the quantitative hemolysis assay resulted in OD reads of 1.22 ± 0.11, 1.17 ± 0.09, and 1.12 ± 0.10 after rabbit red blood cells (rRBCs) being exposed to the fungal concentrations of 10⁴, 10⁵, and 10⁶ spores/mL, respectively, whereas the positive and negative controls had absorbances of 5.55 ± 0.96 and 1.05 ± 0.10, respectively. No differences were identified between the absorbances obtained for microplate wells containing rRBCs and each fungal concentration (*P* = 0.95 between 10⁶ and 10⁵ spores/mL; *P* = 0.88 between 10⁶ and 10⁴ spores/mL; and *P* = 0.93 between 10⁵ and 10⁴ spores/mL), as well as between these wells and negative control (*P* = 0.91, 0.86, and 0.79, for comparisons between the negative control and the wells containing rRBCs and 10⁶, 10⁵, and 10⁴ spores/mL, respectively). Moreover, the mean absorbance in the positive control was significantly higher than in wells containing rRBCs and each fungal concentration and the negative control (*P* < 0.01 for all comparisons) (Fig. 2). Thus, aggregated results from these qualitative and quantitative assays confirm that *M. circinelloides* tested negative for hemolysin production.

### Impact of *M. circinelloides* in the avian intestinal micro- and mycobiome and homeostasis

**Fig 3:**
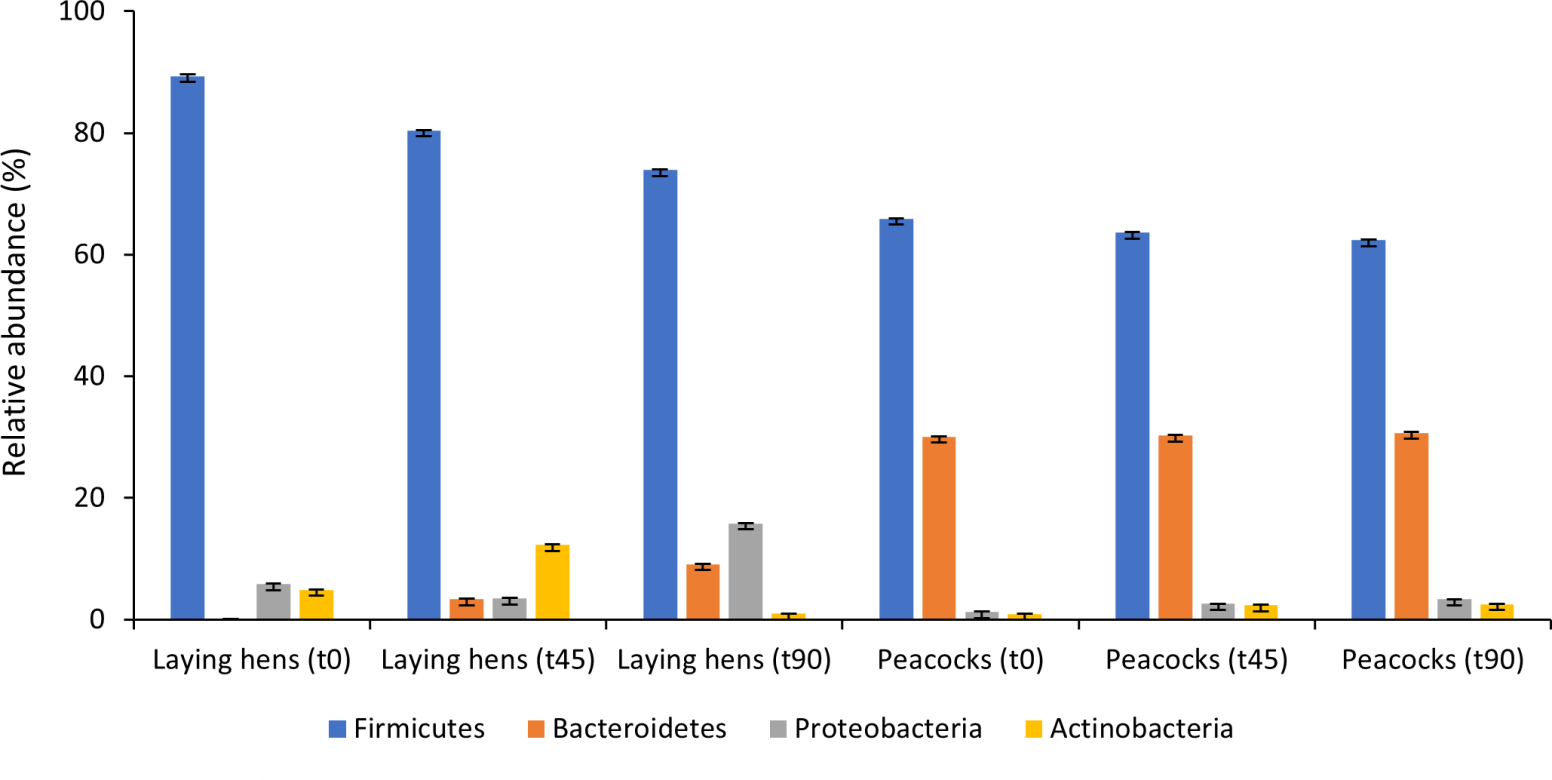
Dynamics of native GI bacteria phyla during the *in vivo* trials in laying hens and peacocks following *M. circinelloides* spores’ administrations.

**Fig 4:**
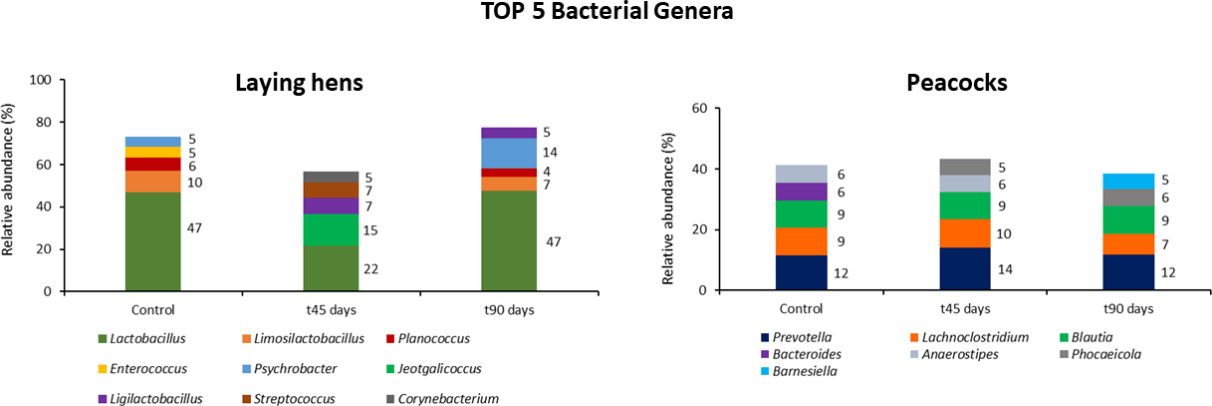
Top five bacterial genera in each fecal sampling time point, measured by relative abundance, following the administration of *M. circinelloides* spores to laying hens and peacocks.

Fecal microbiome (i.e., bacteria) sequencing revealed that bacteria of the phyla Firmicutes (74% ± 0.05%–89% ± 0.34%) and Proteobacteria (4% ± 0.1%–16% ± 0.1%) were the most abundant in the GI tract of laying hens, with the genus *Lactobacillus* is the most represented in the entire study (22% ± 0.1%–48% ± 0.04%) (Fig. 3). Guts from peacocks were mainly colonized by Firmicutes (62% ± 0.1%–66% ± 0.1%) and Bacteroidetes (30% ± 0.1%–31% ± 0.1%), and *Prevotella* spp., *Lachnoclostridium* spp., and *Blautia* spp. were the most abundant genera (Fig. 4). Overall, bacterial phyla and genera relative abundances remained similar during both *in vivo* trials after birds were fed with *M. circinelloides* spores, and the aggregated alpha-diversity for laying hens and peacocks did not differ between each sampling time point (*P* = 0.62).

**Fig 5:**
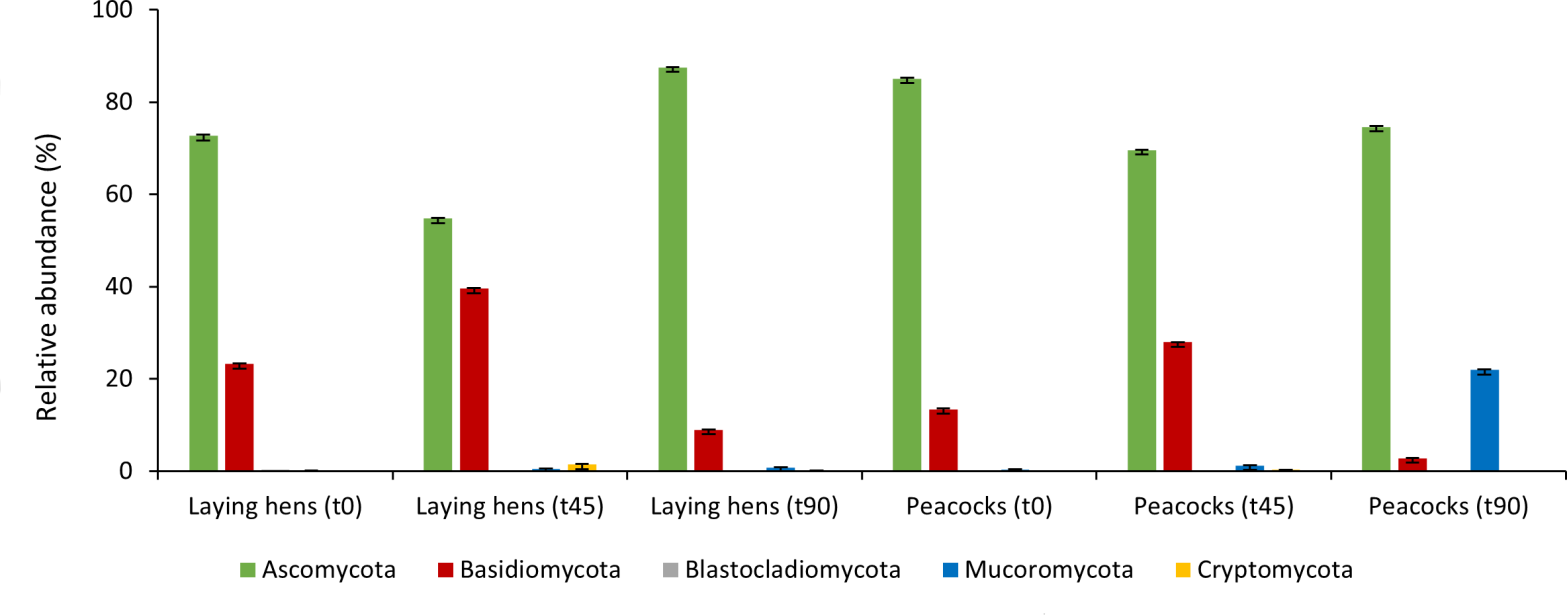
Dynamics of native GI fungi phyla during the *in vivo* trials in laying hens and peacocks following *M. circinelloides* spores’ administrations.

**Fig 6:**
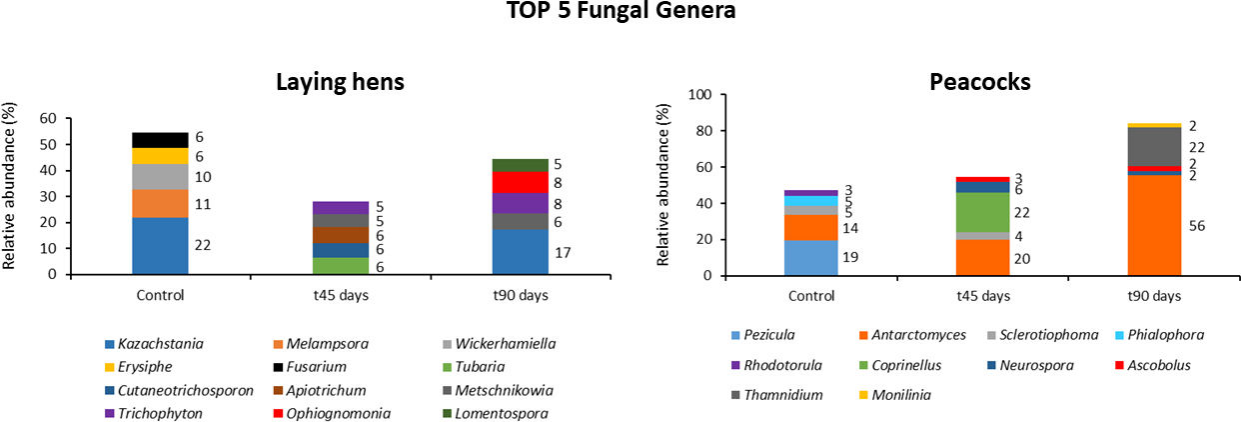
Top five fungal genera in each fecal sampling time point, measured by relative abundance, following the administration of *M. circinelloides* spores to laying hens and peacocks.

The gut mycobiomes of laying hens and peacocks were found to be dominated by fungi of the phyla Ascomycota and Basidiomycota (55% ± 0.1%–88% ± 0.1%), with the exception of peacocks after 90 days of fungal administrations, in which Mucoromycota was the second most abundant phylum (22% ± 0.1%) (Fig. 5). Also, gut fungal genera composition differed between laying hens and peacocks, with the feces of the first being mainly composed of fungi of the genus *Kazachstania* at the beginning and end of the *in vivo* trial (22% ± 0.1% and 17% ± 0.04%), whereas *Pezicula*, *Coprinellus,* and *Antarctomyces* were the most abundant genera in the GI tract of peacocks at t0, t45, and t90 days (19.5% ± 0.2%, 21.96% ± 0.003%, and 55.7% ± 0.1%, respectively) (Fig. 6). Moreover, no *M. circinelloides* sequences were detected in feces from both bird collections in every trial’s time point. As observed for the microbiome, the fungal diversity was not affected by the administration of *M. circinelloides* spores, as the aggregated alpha-diversity did not differ between each sampling time point (*P* = 0.15).

Moreover, fecal appearance analysis revealed that laying hens’ intestinal homeostasis seemed to remain constant during the *in vivo* trial, with 70%–100% of the samples showing normal appearance. In fact, a significant increase in the proportion of normal feces after 45 days of fungal feeding was recorded (100%, *P* = 0.02) in comparison with those from the control time point. Also, 88%–100% of peacock fecal samples were classified as normal regarding their appearance and consistency, never differing significantly from those of the control time point (Table 1). Finally, birds exhibited normal behavior, and no changes were observed on skin and feathers throughout the trials.

**TABLE 1:** Intestinal homeostasis evaluation performed during the assays in laying hens and peacocks, measured by the prevalence of normal fecal samples in comparison with diarrheic or hemorrhagic feces^*a*^

Time point	Laying hens					Peacocks				
	N	D	H	% N (N/T)	*P*-value	N	D	H	% N (N/T)	*P*-value
Control	14	3	3	70	NA^*b*^	15	2	0	88	NA
T15 days	18	0	2	90	0.235	19	1	0	95	0.58
T30 days	16	3	1	80	0.716	15	1	0	94	0.52
T45 days	20	0	0	100^*c*^	0.02	20	0	0	100	0.20
T60 days	17	2	1	85	0.45	20	0	0	100	0.20
T75 days	18	0	2	90	0.235	20	0	0	100	0.20
T90 days	19	0	1	95	0.09	20	0	0	100	0.20

^*a*^ N, normal feces; D, diarrheic feces; H, hemorrhagic feces; and T, total (N + D + H).

^*b*^ NA - Not applicable.

^*c*^ Significant differences in comparison with the control time point (*P* < 0.05).

## DISCUSSION

Research with ovicidal and larvicidal fungi has revealed promising results for their use in the biological control of avian GI parasites, namely coccidia, ascarids, and strongyles (4, 15, 16). Despite their demonstrated efficacy in destroying parasitic forms, ensuring the safety of these fungi for animals and technicians is a crucial step when choosing the most suitable fungal strains for parasite management in farms, zoos, and public/private animal collections.

The whole-genome sequencing (WGS) of this *M. circinelloides* isolate allowed to identify six predicted genes coding for virulence factors, namely an iron permease (FTR1), iron receptors (FOB1 and FOB2), ARFs (ARF2 ARF6), as well as a Rho-like GTPase (CDC42). As iron is essential for fungal survival, the permease FTR1 and iron receptors FOB1 and FOB2 play an important role in free iron uptake and, thus, in the virulence of Mucorales fungi, especially when the proteins lactoferrin and transferrin fail to chelate the free iron available in host tissue fluids as a consequence of altered metabolism, lysis of red blood cells, or even trauma (17, 18). Also, studies have been demonstrating that ARFs play an essential role in *M. circinelloides* dimorphism and virulence, such as by regulating the biogenesis of vesicles involved in hyphal apical growth (18, 19). Moreover, the GTPase CDC42 is essential for hyphae morphogenesis in filamentous fungi due to its role in the generation of cell polarity (20), and its coding gene has also been identified in the genome *P. chlamydosporia*, another ovicidal fungus species. In this particular case, the detection of the CDC42 gene can also be seen as an important attribute of this *M. circinelloides* isolate since hyphae germination and migration toward parasite eggs and oocysts is the first step of the parasiticide activity developed by this kind of fungi (2, 4).

Also, regarding the phenotypic expression of virulence factors, the current study showed that the FMV-FR1 *M. circinelloides* isolate only tested positive for lecithinase activity. This enzyme hydrolyzes the phospholipid lecithin, which is a structural component of the animal cell membrane, and thus might play a role in fungal pathogenicity and eventually lead to host cell lysis (21). Negative results for hemolysin and biofilm assays are in contrast with previous research performed in other medically important fungi, namely *Aspergillus*, *Candida*, *Fusarium*, *Cryptococcus*, and *Coccidioides*, which reported these fungal taxa as being hemolysin producers and having biofilm-forming abilities in abiotic and biotic substrates (22, 23).

In the second phase of the present study, the analysis of birds’ gut microbial communities was performed on fresh fecal samples from both avian hosts, collected immediately after excretion. This is an easy, non-invasive, and accurate approach for estimating the overall bacterial and fungal communities in animals’ GI tract by sequencing their fecal microbiome and mycobiome (12). Fecal microbiome sequencing results from laying hens revealed that their guts were overall colonized by bacteria of the phyla Firmicutes, followed by Proteobacteria, and the genus *Lactobacillus* had the highest relative abundance, whereas the GI microbiota of peacocks was mostly dominated by Firmicutes and Bacteroidetes. These results are in accordance with Grond et al. (12) and Diaz Carrasco et al. (24), who reported that in physiological conditions, the native GI microbiota of most bird species, namely Galliformes, is mainly dominated by Firmicutes and described the crucial role of these bacterial communities in the biosynthesis of short-chain fatty acids (byproducts of fermentation), which are important energy and carbon sources for bird’s nutrition. The abundance of some bacterial taxa belonging to this phylum, namely *Lactobacillus* spp., has been positively associated with higher productivity in poultry (24). Also, *Lactobacillus* spp. and *Bacillus subtilis* are examples of bacteria with probiotic properties, contributing to the regulation of birds’ gut microbiota toward higher proportions of beneficial bacteria and thus improving the animals’ intestinal homeostasis and nutrient uptake (24, 25). Animals’ GI microbiota is influenced by several biotic and abiotic factors, and the identification of the Bacteroidetes phylum as the second most abundant in peacocks during the entire trial and with similar relative abundances at every sampling time point might be related to the feed composition, which contained corn grains and sunflower seeds, among other constituents. The presence of these components in feed may have stimulated the growth of bacteria from this phylum since they play an important role in degrading complex plant polysaccharides, such as cellulose (12).

The phyla Ascomycota and Basidiomycota were the most abundant in the GI tract of laying hens and peacocks throughout the assays, with the exception of the last fecal sampling time point in peacocks, in which fungi of the phylum Mucoromycota were the second most abundant after Ascomycota. Although most studies regarding the characterization of fungal intestinal communities have been performed in humans and rodents, recent studies revealed that the GI mycobiota of chickens is mainly colonized by Ascomycota and, in less extension, by Basidiomycota fungi, irrespective of the GI tract’s section (14), which is in accordance with the current study results. Also, yeasts of the genus *Kazachstania* were identified as the most abundant at two sampling time points (beginning and end of the trial) in laying hens, being a genus highly represented in the GI tract of other monogastric species, such as pigs, and with a reported positive role in promoting intestinal epithelial glycolysis (26).

The increase in the relative abundance of the Mucoromycota phylum in peacock feces, at the end of the *in vivo* trial, is another interesting result observed in this study. The last weeks of this assay were marked by flood episodes in Lisbon downtown, as a result of intense rainfall, which led the birds’ owners to house them together. Mucorales fungi are known for their ubiquity and rapidly growing mycelium (27), and thus birds’ stocking conditions observed for that period might have increased fungal spores’ transmission through the fecal-oral route, consequently being responsible for a higher fecal excretion of Mucorales spores at the end of the trial.

Also, mycobiome sequencing failed to specifically detect *M. circinelloides* sequences in feces from laying hens and peacocks in each sampling time point, suggesting that spores from this fungus were not capable of colonizing the GI tract of birds. Although further anatomopathological analysis is needed to confirm this hypothesis in birds, the incapability of *M. circinelloides* to colonize the GI tract of other animal species has already been demonstrated in a previous study in ruminants (28).

The overall results obtained in each bird flock suggest that feeding *M. circinelloides* spores to laying hens and peacocks did not alter their GI microbiome and mycobiome, with *Lactobacillus* spp. and *Prevotella* spp. being the top bacterial genera in all sampling time points, in laying hens and peacocks, while the fungal genus *Kazachstania* was the most abundant in laying hens’ feces at the beginning and end of the trial. Some fluctuations were still observed in microbial phyla and genera throughout the study, which could have been influenced by several biotic and abiotic factors. For example, age is a biological factor that has been shown to influence birds’ gut microbial composition, with previous studies in chickens revealing that the cecum of newly hatched chicks is mainly dominated by Clostridiaceae, whereas *Lactobacillus* represents 25% of all cecal bacterial genera at three days of age and then its abundance decreases up to 100 times when broilers reach 42 days (29–31). However, there are still few publications addressing the interactions between age evolution in animal groups and their gut microbial composition and diversity, especially for mycobiome, with a previous study reporting the transition from a *Trichosporon* spp. dominance in the cecum of broilers aging 14 days to *Microascus* spp. at 28 days of age, despite no difference being recorded in the fungal alpha-diversity (32). Moreover, some shifts observed in the fecal microbiome and mycobiome analysis may have been caused by the intrinsic instability of the fecal bacterial and fungal communities, which rapidly change their relative abundances depending on storage conditions (30). These limitations of the current study suggest that further research should include a previous separation of birds into two distinct groups, with the control group receiving feed not supplemented with fungal spores, as well as more fecal samplings to cover a broader timeframe of analysis.

Despite the identification of several predicted genes in the *M. circinelloides* (FMV-FR1) genome coding for virulence factors and a positive lecithinase activity, overall results from this virulence profile analysis, combined with the lack of interference of *M. circinelloides* spores in birds’ gut microbial diversities and the absence of alterations in birds’ fecal appearance and consistency, allow to have an initial insight on the lack of pathogenicity of this *M. circinelloides* isolate to birds. The absence of differences in the bacterial and fungal alpha-diversities, after birds are fed with *M. circinelloides* spores, is another interesting result from the current study, as microbial equilibrium is essential for intestinal homeostasis (30, 33).

Parasiticide fungi have always been tested on animals in controlled health programs, with constant monitoring of any eventual side effects developed by the animals. Previous *in vivo* studies in which parasitized animals received *M. circinelloides* (CECT 20824) spores found that their hematological parameters (e.g., red blood cells, hemoglobin, hematocrit, white blood cells, and lymphocytes) remained constant or even improved after fungus administrations (7, 34). Also, the anatomopathological analysis of different dairy cow tissues revealed no signs of damage caused by *M. circinelloides* (CECT 20824) and *D. flagrans* (CECT 20823) spores (28).

To the best of our knowledge, this study represents the first report regarding the analysis of the virulence profile of an *M. circinelloides* parasiticide isolate and the assessment of its potential impact on animals’ GI core bacterial and fungal communities. Despite overall results suggesting that this *M. circinelloides* isolate does not offer any health risk for birds, in the scope of a parasite biological control program, more *in vitro* and *in vivo* studies are needed to confirm this hypothesis, namely assessing the phenotypic expression of more potential virulence factors, collecting blood samples for hematological analysis, performing anatomopathological analysis in birds receiving spores of *M. circinelloides* FMV-FR1, and finally integrating the microbial results with parasitological data by comparing the dynamics of the gut microbiome and mycobiome following the reduction of the GI parasitic population caused by the administration of fungi with parasiticidal properties.

## MATERIALS AND METHODS

### Fungal isolate

This study focused on an *M. circinelloides* (FMV-FR1) isolate belonging to the Laboratory of Parasitology and Parasitic Diseases of the Faculty of Veterinary Medicine, University of Lisbon (LPPD-FMV), with previously demonstrated parasiticide activity toward avian coccidia (4). This isolate was stored in wheat-flour agar medium (WFA, 2%) at room temperature and in a wheat broth (10 grams of wheat grains per 1 L of distilled water). This broth was previously autoclaved and transferred to sterilized plastic bottles, after which it was inoculated with WFA cubes of 2.25 × 2.25 × 2.25 cm, containing mycelia from *M. circinelloides*, and then left at room temperature for 1 month with a slope of 45° (35).

Fungal mycelium from the first formulation was used in the first phase of this study (virulence profile assessment), whereas the wheat broth enriched with fungal spores was used in the second phase (*in vivo* trials).

### Phase 1—assessing *M. circinelloides* virulence profile

#### Whole-genome sequencing

The extraction of *M. circinelloides* DNA was done by phenol/chloroform followed by precipitation with sodium acetate and ethanol, and finally resuspended in Tris-EDTA buffer (36). The obtained DNA was purified using AMPure XP beads (Beckman Coulter, High Wycombe, UK), and its quality and concentration were assessed by NanoDrop One and Qubit 4 Fluorometer (Thermo Fisher Scientific Inc., Waltham, USA).

The isolate’s genomic DNA was subjected to WGS using Oxford Nanopore PromethION (P24), with R10.4.1 flow cell (FLO-PRO114M) and Ligation Sequencing Kit V14 (SQK-LSK114) following the manufacturer’s instructions (Oxford Nanopore Technologies, Oxford, UK). After 17 h, sequencing yielded 1,950,000 long read sequences with a size *N*_50_ of 18 Kb. A total of 21.27 Gb of data were produced (500× genome coverage). Raw reads were classified by MinKNOW (Oxford Nanopore Technologies, Oxford, UK) based on the average read quality score > 7 and then assembled using the pipeline Canu (version 2.2), with the parameters “genomeSize = 39 mb” and “-nanopore.” The output FASTA file from Canu was polished using the tool Medaka (version 1.6.0), with the parameter “-m r103_hac_g507.”

The pipeline Funannotate (https://github.com/nextgenusfs/funannotate) was used to explain the assembled genome with the following commands: funannotate clean; funannotate sort; funannotate mask; funannotate predict with the parameter “*Mucor racemosus”*; funannotate iprscan, using the pipeline InterProScan (version 5.61-93.0), and funannotate annotate. Gene IDs recorded correspond to hypothetical genes given by the Funannotate pipeline.

Protein sequences associated with fungal pathogenicity were retrieved from the Database of Fungal Virulence Factors (DFVF, http://sysbio.unl.edu/DFVF/Download/AllGenes.txt) (37) and Pathogen-Host Interactions Database (PHI-BASE, https://www.phi-base.org) (38). Protein sequences generated by the Funannotate pipeline were used to perform a BlastP against DFVF and PHI-BASE databases, using a local instance of SequenceServer 2.0.0, BLASTP 2.12.0+, and with the following parameters: *e*-value 10^−6^, matrix BLOSUM62, gap-open 11, gap-extend 1, and filter F (39). Cut-offs equal to 10^−6^ for *e*-value and >80% for genes identity were chosen based on the Funannotate pipeline, Chaudhuri and Ramachandran (40), and on the standardized procedures of the Biosystems and Integrative Sciences Institute of the Faculty of Sciences, University of Lisbon.

#### Phenotypic expression of virulence factors

This step aimed to assess the phenotypic expression of common six microbial virulence factors by the *M. circinelloides* isolate (FMV-FR1), namely the enzymes proteinase, lecithinase, DNase, gelatinase, and hemolysin, and also biofilm production. All media, incubation conditions, and expected outcomes for each virulence factor are summarized in Table 2. The analysis of the phenotypic expression of all virulence factors was performed by direct visualization of each plate and following all procedures from previous research by Cunha et al. (41) and Raposo et al. (42) and their control plates.

**TABLE 2:** List of virulence factors assessed in *M. circinelloides* (FMV-FR1), culture media and incubation conditions used for this purpose, and expected phenotypic expression

Virulence factors	Media	Incubation conditions	Outcomes
Proteinase	Skim milk medium: skim milk powder and bacteriological agar (VWR, Leuven, Belgium)	26°C for 72 h	Positive result—appearance of a clear zone surrounding the colonies
Lecithinase	Tryptic Soy agar supplemented with 10% egg yolk emulsion (VWR, Leuven, Belgium)		Positive result—appearance of a white precipitate around the colonies
DNase	DNase medium (Thermo Fisher Scientific–Remel, Lenexa, USA) supplemented with 0.01% toluidine blue (Merck, Darmstadt, Germany)		Positive result—appearance of pink halos around the colonies
Gelatinase	Nutrient Gelatine agar (Oxoid, Hampshire, UK)		Positive result—cumulative effect of gelatine liquefaction after incubation and maintenance of its liquid consistency after cooling at 4°C for 30 minutes
Hemolysin	Columbia agar supplemented with 5% sheep blood (bioMérieux, Marcy-l’Etoile, France)		α-Hemolysis (partial activity)—appearance of green halos around the colonies β-Hemolysis (full activity)—clearing halos Negative result—absence of halos
Biofilm production	Red Congo agar: Brain-Hearth Infusion Broth (VWR, Leuven, Belgium), bacteriological agar (VWR, Leuven, Belgium), and Red Congo reagent (Sigma-Aldrich, Steinheim, Germany)		Positive result—black colored medium around the colonies: Strong producer: after 24 h Medium producer: after 48 h Weak producer: after 72 h

Moreover, a second experiment was performed aiming at quantifying the rate of red blood cell destruction by measuring the absorbance of hemoglobin (Hb) release after exposure to fungal spores. Two blood samples were collected from two different male lionhead rabbits younger than 1-year old on the day before the experiment and during routine clinic consultations at “Exoclinic” (Lisbon, Portugal). Blood was taken from the lateral saphenous vein into EDTA tubes and stored in a refrigerator at 4°C. Then, a protocol adapted from Mendonça et al. (43) was applied. For each blood sample, a total of 1 mL of blood was centrifuged at 4,000 rpm, for 5 minutes, and the resulting pellet was washed two times in 1× phosphate-buffered saline (PBS) solution (150 mM NaCl, 5 mM Na_2_HPO_4_, and 1.7 mM KH_2_PO_4_; pH 7.4) by centrifugation at 4,000 rpm for 5 minutes, until obtaining a clear supernatant. Then, the supernatant was discharged, and the pellet containing rRBCs was resuspended in PBS solution to a final concentration of 0.5% (vol/vol). *M. circinelloides* suspension with an average concentration of 6.04 × 10^6^ spores/mL was also prepared in PBS solution by using a Neubauer chamber to count fungal conidia. Serial dilutions (1:2) were performed in microplates, with test wells containing 100 µL of each fungus concentration and 100 µL of rRBCs’ suspension, corresponding to a final fungal concentration ranging between 10^4^ and 10^6^ spores/mL. Positive and negative controls were also performed (100 µL of Triton (1%) and 100 µL of rRBCs’ suspension, or only 200 µL of PBS, respectively). Microplates were incubated at 37°C for 1 h with 100 rpm stirring and then centrifuged at 4,000 rpm at 4°C for 5 minutes. Supernatants were then transferred to a new microplate, and hemolysis was determined by Hb release, measured by absorbance at 450 nm using a FLUOstar OPTIMA microplate reader (BMG LABTECH, Offenburg, Germany) and the software BMG LABTECH OPTIMA (version 2.20 R14). In this experiment, each blood sample evaluation was performed in three repetitions, each with two replicates.

### Phase 2—effect of *M. circinelloides* spores in avian native gut bacteria and fungi

#### Domestic and exotic bird collections

Between July and December 2022, two *in vivo* trials were performed in two different avian collections located in the Lisbon district, Portugal: a laying hens’ flock (*Gallus gallus domesticus*) from a livestock farm (39°13′54.373″ N 9°17′2.235″ W) and a peacock flock (*Pavo cristatus*) from São Jorge Castle (38°42′50.241″ N 9°8′2.182″ W).

The trial with laying hens was performed between July and September 2022. The flock was composed of 100 birds, 1-year old, kept under free-range conditions, with an outdoor area of 625 m^2^ and a housing of 45 m^2^ with feeders, water drinkers, perches, and nests. The trial with peacocks was performed between October and December 2022, with the flock composed of 58 birds (44 adults and 14 juveniles), free ranging in an outdoor area of 4,700 m^2^.

According to the routine practices performed in each bird collection, laying hens and peacocks were fed with avian commercial feed once or twice per day, respectively. Also, both bird flocks were not subjected to any antimicrobial drug treatment for at least 6 months prior to and during the trials.

#### Fungal formulations

*M. circinelloides* spores’ suspensions in wheat broth were added to feeders at farm level prior to feeding birds. This approach was chosen due to the poor climatization of the farm’s storage room, which would contribute to the rapid growth of fungi on feed and eventually to its rejection by the animals.

The procedure was adapted from the protocol described by Palomero et al. (9). Two hand sprayers were filled with the *M. circinelloides* suspension (10⁶ spores/mL), which was then sprayed on feed after being placed in feeders. Laying hens received a dose of 6.8 × 10^7^ spores/kg of feed in each administration time point.

For peacocks, the administration procedure was slightly different. Bird feed doses enriched with *M. circinelloides* spores were previously prepared in the LPPD-FMV, adapting the procedures reported by Voinot et al. (7). Each formulation was composed of 600 grams of bird feed mixed with 60 mL of fungal suspension, which was dried at 27°C for 30 minutes using an incubator. Individual doses were prepared in sealed plastic bags, and peacocks received 1.01 × 10^8^ spores/kg of feed at each administration time point.

#### Experimental design

**Fig 7:**
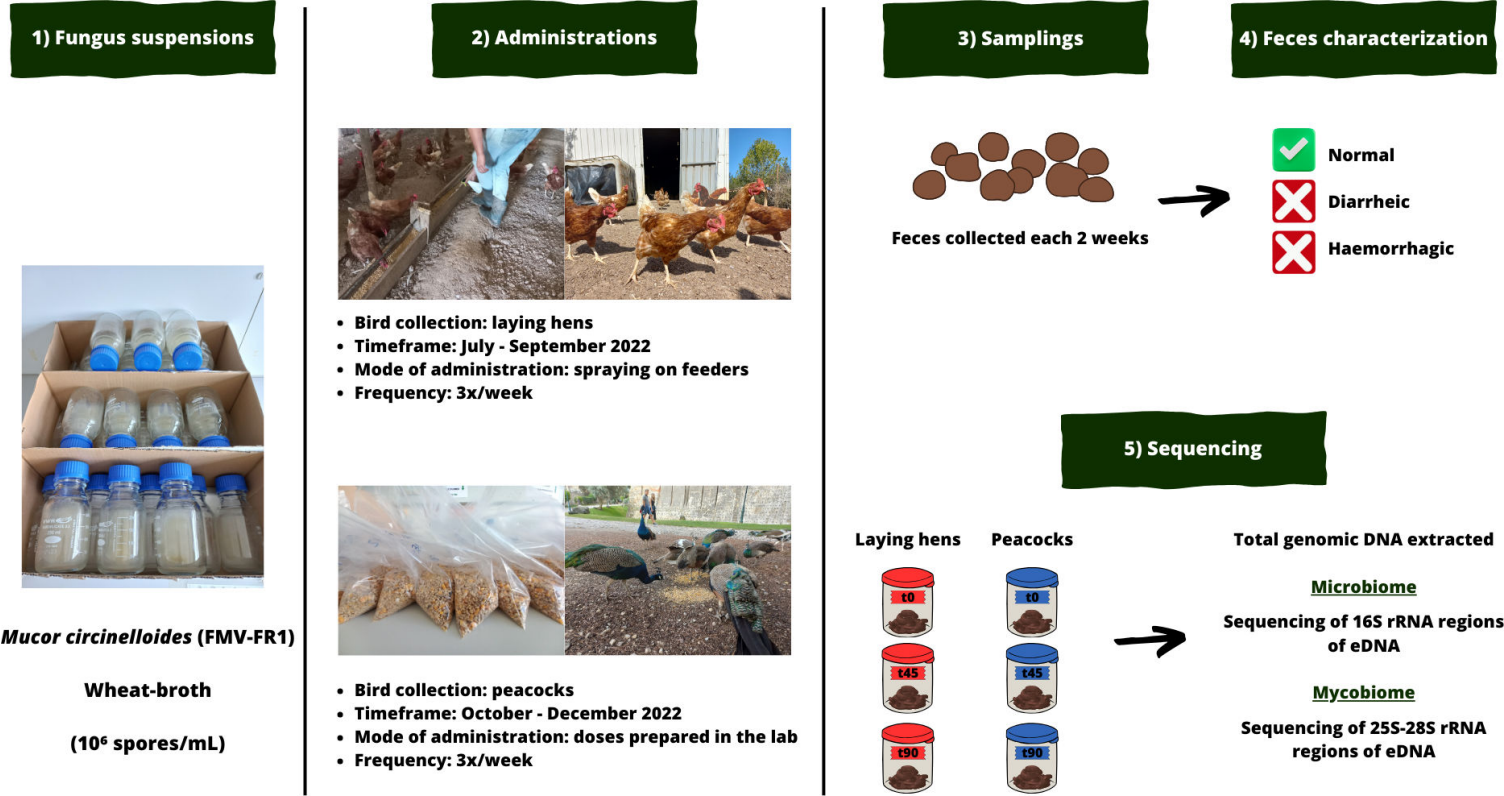
Experimental design established for assessing the effect of *M. circinelloides* (FMV-FR1) spores on birds’ feces appearance and consistency and on their fecal microbiome and mycobiome (figure created using Canva, https://www.canva.com; photos from fungal suspensions, bird flocks, and mode of administration are original).

In both bird collections, a total of 33 fungus administrations were performed *per os* three times a week. Then, the number of samples collected on each bird flock was determined based on the availability of fresh feces at the control time point in laying hens, 20 samples, in which the first trial was performed. Thus, the same quantity of samples was collected at further sampling time points, each lasting 2 weeks, for 3 months, and in both bird flocks, with the only exceptions being observed for the control and t30-day time points in peacocks, in which it was only possible to collect 17 and 16 fresh fecal samples, respectively. Since it was not possible to establish two separate groups (test and control) in each bird flock, the initial samples served as a control (t0 days), whereas samples from t15 to t90 days corresponded to the test phase, as also described by Paz-Silva et al. (10) (Fig. 7). Feces were packed in individual plastic bags and immediately transported in a cooling bag to the LPPD-FMV.

**Fig 8:**
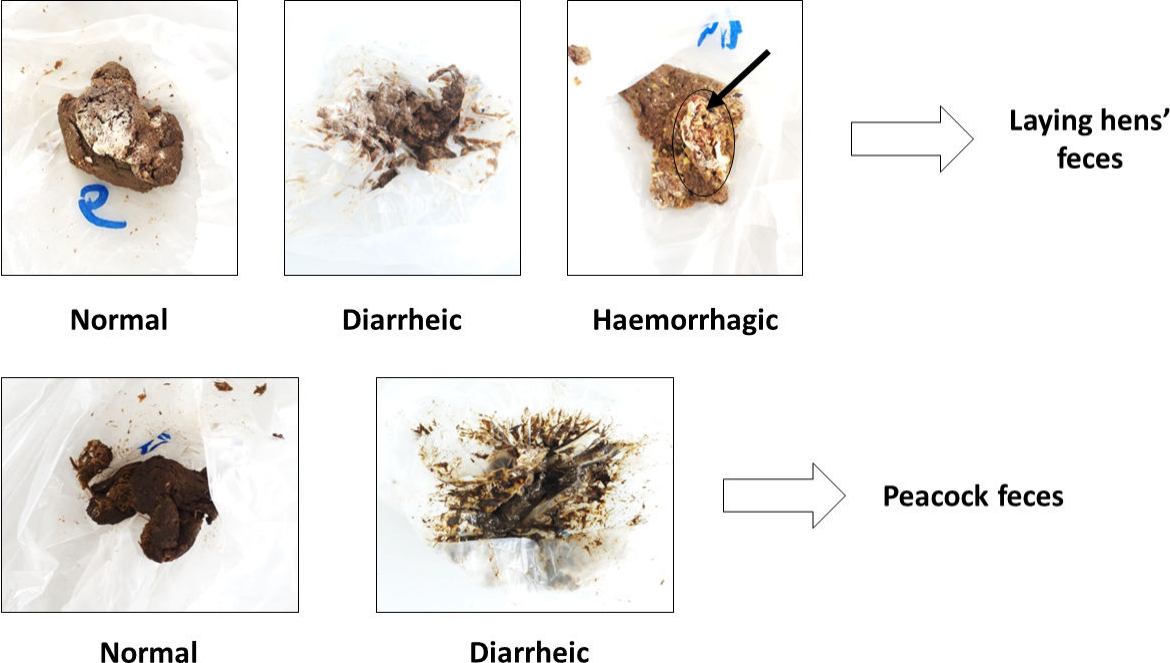
Qualitative scale used for fecal samples’ appearance and consistency characterization (normal vs diarrheic or hemorrhagic); black circle and arrow highlight the blood clot identified in a laying hen’s fecal sample (original photos).

In each sampling time point and in both bird flocks, fecal samples’ appearance (normal vs hemorrhagic) and consistency (normal vs diarrheic) were analyzed (Fig. 8), with the number of samples in each category being quantified and compared with those from the control time point. Then, all feces from the control, t45, and t90 days time points were gently mixed and homogenized, forming three aggregated sub-samples per bird flock, which were then placed in sterile plastic flasks and stored in a freezer (−20°C) until further processing. Since *in vivo* trials were performed in bird groups, the procedure of aggregating feces in each sampling time point aimed to reduce the variability between individuals and thus establish sub-samples that would be more representative of the overall flocks’ resident bacterial and fungal communities.

#### Microbiome and mycobiome sequencing

Total genomic DNA was extracted from all aggregated sub-samples using the DNeasy PowerMax Soil Kit (QIAGEN, Venlo, the Netherlands), following the manufacturer’s instructions.

Each sample was subjected to microbiome and mycobiome characterization (bacterial and fungal sequences, respectively) by sequencing the 16S and 25S-28S rRNA regions of eDNA. Samples were analyzed by a customized analytical pipeline developed by BioISIGenomics for long-read targeted nanopore sequencing to obtain high-accuracy taxonomical classification. The current approach has been validated through ZymoBIOMICS Microbial Community Standard, and sequencing runs were carried out on the GridION X5 sequencing platform. Sequencing data were obtained from 16S and 25S-28S rRNA amplicons, low-quality reads were removed, and the remaining reads were size selected (keeping reads between 1,200 and 1,700 bp) using prinseq-lite (44). Taxonomic classification was performed using a lowest common ancestor approach, with indexing based on k-mers mapping to the lowest common ancestor of all genomes known to contain a given k-mer (45). Following classification, data were rarefied and subjected to phyla and genera relative abundance analysis (percentage of each phylum or genus reads in the total of raw reads) as well as alpha-diversity group analysis based on calculating the Shannon diversity (46) and Pielou evenness (47) indexes using the Qiime2 software (version 2019.4.0) (48). Three technical replicates were performed for each aggregated sub-sample (nine technical replicates for each bird flock).

### Statistical analysis

Data regarding the quantitative hemolysis assay were subjected to descriptive analysis (mean and standard error values) using the software IBM SPSS Statistics, version 27 (IBM Corporation, Armonk, NY, USA). Also, a normality analysis was performed using the Shapiro-Wilk test (*n* < 50), and it was concluded that the OD results obtained for wells containing rRBCs and each fungal concentration and the positive and negative controls were normally distributed (10^4^ spores/mL: *P* = 0.20; 10^5^ spores/mL: *P* = 0.13; 10^6^ spores/mL: *P* = 0.10; positive control: *P* = 0.19; and negative control: *P* = 0.31). Thus, the one-way ANOVA with *post hoc* LSD test was used to compare the results between these five groups of OD data. Moreover, fecal appearance and consistency were compared between each trial’s time point in both bird flocks using the Fisher’s Exact test.

Microbiome and mycobiome data were treated using the software R, version 4.1.2 (The R Foundation, https://www.r-project.org/foundation/). Alpha-diversity group analysis was subjected to the Kruskal-Wallis test to compare bacterial and fungal fecal diversities between each sampling time point. A significance level of *P* < 0.05 was used for every statistical test.

## Data Availability

Whole-genome sequencing data recorded for *Mucor circinelloides* isolate FMV-FR1 have been uploaded to the NCBI database with the BioProject accession code “PRJNA1065632” and genome submission code “SUB14153967.”

## SUPPLEMENTAL MATERIAL

Table S1 spectrum.04078-23-s0001.xlsx List of hypothetical genes coding for virulence factors, after WGS of the tested *M. circinelloides* isolate.

ASM does not own the copyrights to Supplemental Material that may be linked to, or accessed through, an article. The authors have granted ASM a non-exclusive, world-wide license to publish the Supplemental Material files. Please contact the corresponding author directly for reuse.
